# Influence of Carbon Quantum Dots on the Orientational Order and Rotational Viscosity of 8CB

**DOI:** 10.3390/nano15161278

**Published:** 2025-08-19

**Authors:** Alfredos Schinas, Stefanos Basim Atata, Dimitris Tsiourvas, Ioannis Lelidis

**Affiliations:** 1 Faculty of Physics, National and Kapodistrian University of Athens, Panepistimiopolis, Zografos, 15784 Athens, Greece; freddie@phys.uoa.gr (A.S.);; 2 Institute of Nanoscience and Nanotechnology, National Centre for Scientific Research Demokritos, Aghia Paraskevi, 15310 Athens, Greece; d.tsiourvas@inn.demokritos.gr

**Keywords:** soft nanocomposites, liquid crystals, quantum dots, birefringence, Fréedericksz transition, switching times, viscosity, activation energy

## Abstract

Soft nanocomposites were prepared by dispersing lipophilic carbon quantum dots (CQDs) in the liquid crystal compound 8CB. The quality of the dispersion was evaluated using fluorescence microscopy, while the microstructure of the samples was examined via polarized optical microscopy. We investigated the influence of CQDs on the orientational order parameter *S* as a function of temperature and sample composition by measuring birefringence. Additionally, the Fréedericksz transition threshold, along with the characteristic response and relaxation times, was measured for each sample as a function of temperature and applied voltage amplitude. The extracted rotational viscosity $\gamma_{1}$ exhibits a pretransitional divergence upon cooling toward the smectic-A phase. Its temperature dependence was analyzed using established models from the literature, and the corresponding activation energy was determined. Notably, our analysis suggests that the presence of CQDs alters the power-law dependence of $\gamma_{1}$ on the orientational order parameter *S*. The influence of CQDs on the elastic constants has been investigated.

## 1. Introduction

Nematic liquid crystals (NLCs) are birefringent structured fluids with high sensitivity to relatively weak external fields. They are used in a wide range of applications, including flat-panel displays, optical filters, optical valves, flow generation, lasing, optical switches for telecommunications, and directional reflectors. In calamitic nematics, molecules align, on average, along a common direction defined by the nematic director n ($\left|n\right|=1$) [1,2]. The viscosity of nematics depends on the orientation of the nematic director relative to the velocity and its gradient. The coupling between flow and orientation was analyzed in [3,4,5,6]. Moreover, viscosity is influenced by molecular structure, intermolecular interactions, and temperature. A key factor determining the response speed of liquid crystal displays and other devices is rotational viscosity. Rotational viscosity describes the internal friction experienced by mesogenic molecules rotating around an axis perpendicular to the director. It is quantified by the rotational viscosity coefficient, $\gamma_{1}$. The electro-optical switching times of an NLC device in response to an external field, which involves orientational transitions of the nematic configuration, depend linearly on $\gamma_{1}$ [7].

Several models have been developed to describe rotational viscosity, its origin, and its dependence on parameters such as temperature (T) and orientational order (S). These models include the Diogo and Martins model, which integrates several earlier models [8,9], the microscopic theory of Osipov and Terentjev [10], the kinetic approach of Doi [11], and the geometric approach [12,13]. Further, rotational viscosity was investigated by molecular dynamics calculations [14,15,16]. Deviations of experimental data from theoretical predictions are common [17,18,19]. A common problem when using these models to fit experimental data on $\gamma_{1}$ is the presence of more than three fitting parameters, as already noted in [17,20]. Experimentally, $\gamma_{1}$ is typically measured using the optical response time method [20,21,22,23,24]. An alternative technique involves the use of a rotating magnetic field [25,26,27,28]. Finally, a third method, based on transient current measurements, was proposed in [29].

The purpose of the present study is to investigate the impact of carbon quantum dot (CQD) nanoparticles (NPs) on S and $\gamma_{1}$, to determine whether the corresponding activation energy changes in the presence of CQDs, and to verify whether the expected divergence of $\gamma_{1}$ upon cooling towards the smectic-A phase is preserved. To the best of our knowledge, no previous studies have examined this particular soft nanocomposite. Furthermore, the effect of NPs on the divergence of $\gamma_{1}$ has not been investigated. Specifically, we focus on the effect of doping the liquid crystal compound 4-cyano-4′-octylbiphenyl (8CB) with long-alkyl-chain-functionalized CQDs, and we exploit their strong fluorescence to evaluate their dispersion in situ. We conducted static and dynamic electro-optical measurements on both pure and CQD-doped 8CB. From these measurements, we determined S and the voltage threshold for the reorientational Fréedericksz transition in splay geometry cells as functions of temperature and nanoparticle concentration. The rotational viscosity $\gamma_{1}$ was obtained by analyzing the dependence of switching times on temperature and composition using elastic constants and dielectric anisotropy values from the literature. Finally, $\gamma_{1}$ was fitted to extract the activation energy and its dependence on the nematic order. We analyzed our $\gamma_{1}$ data within the framework of the Diogo and Martins model [8], which accounts for the divergence of $\gamma_{1}$.

## 2. Materials and Techniques

### 2.1. Materials

#### 2.1.1. Liquid Crystal

**Figure 1:**
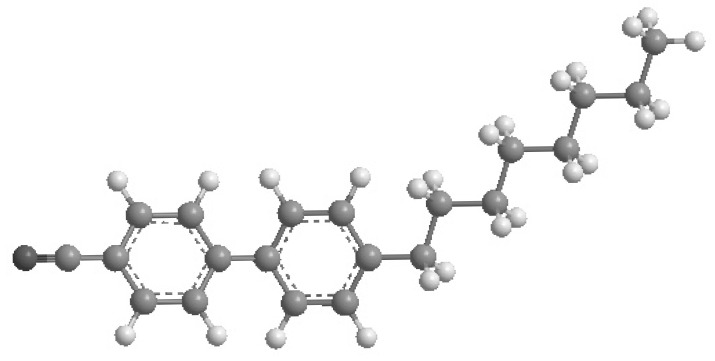
Chemical structure of 8CB.

The liquid crystal compound 8CB (chemical structure shown in Figure 1) was acquired from Frinton Laboratories and used without further purification. The isotropic to nematic transition occurred at $T_{in}=40.12$ °C, while the nematic to smectic-A transition occurred at $T_{na}=33.12$ °C. In addition, 8CB is a well-studied material, and detailed data on its properties are available in the literature; see, for instance, [30,31,32,33,34], among others.

#### 2.1.2. Carbon Nanoparticles

CQDs were synthesized through microwave irradiation of water solutions of citric acid and ethylenediamine, as detailed in [35]. Their characterization via dynamic light scattering (DLS) and transmission electron microscopy (TEM) revealed quasi-spherical, well-separated nanoparticles with diameters ranging from 3 to 6 nm with a mean size of 4.5 nm and a z-potential value of 2.5±1.2 mV. Their maximum fluorescence intensity was observed at 460 nm ($\lambda_{ex}=362$ nm) with a quantum yield of 49%, as reported in [35]. The CQDs were functionalized with C16 chains to minimize intermolecular interactions and improve dispersion in LC phases, avoiding phase separation and aggregation. Taking advantage of the presence of primary amino surface groups of CDs, the functionalization of CQDs with alkyl chains was attained through the interaction of the amino groups of CDs (100 mg) with palmitoyl chloride (1.2 mmol) in dry dimethylformamide (DMF) for 24 h at 40 °C under an inert atmosphere. After precipitation with ether to remove unreacted palmitoyl chloride, followed by drying and further treatment with water to remove unreacted CDs, the obtained dry material was characterized with 1H-NMR in MeOD. Employing naphthalene as an internal standard and comparing the integrations of naphthalene aromatic protons with methyl group protons, it was found that 3.1 mmol of C16 alkyl groups were grafted per 1 g of CDs (see Figure S1). The fluorescence spectra of alkyl-functionalized CDs were obtained in ethanol (see Figure S2) and were, in every respect, the same as those of non-functionalized CDs, with slight changes in their fluorescent maxima, apparently due to the use of MeOH for these measurements instead of the water that was employed for the original CDs. Their excitation spectrum exhibited peaks at 250 nm and at 356 nm, while their emission spectrum revealed a maximum at 446 nm after excitation at 356 nm.

#### 2.1.3. Nanocomposites

The nanocomposite samples were prepared following a solution-based protocol previously described in our earlier works [36,37]. Mixtures were prepared at mass fractions of 0.2% and 4.7% per weight (*w*/*w*). Each mixture was introduced into planar cells with a gap of d=5 μm through capillary suction, at a temperature above the nematic phase.

### 2.2. Techniques

#### 2.2.1. Experimental Setup

Optical observations were performed using a Leica DM2500P polarizing microscope (Leica, Wetzlar, Germany) equipped with a Leica DFC420 digital camera controlled via a PC. The temperature was regulated by an Instec HCS402 (Boulder, CO, USA) heating stage that was mounted on the microscope’s circular stage. The microscope was also equipped with the Fluorescence module Leica SFL4000. The module provided UV light of 365 nm. We utilized it to examine the fluorescence of each sample in reflection mode to assess the dispersion of the CQDs in the liquid crystalline matrix in situ.

The electrooptical response of the sample was measured using the experimental setup outlined in [36]. The microscope is equipped with a photomultiplier tube (Hamamatsu Photonics K.K., H10721, Shizuoka, Japan), which is connected to an oscilloscope (Tektronix, Beaverton, OR, USA, model TDS 2024C). The electric field was applied to every sample cell by a waveform generator (Keithley Instruments, Inc., Cleveland, OH, USA, model 3390) and a voltage amplifier (FLCElectronics, Partille, Sweden, model A400). The wavelength of the incident light was fixed at 546.3 nm through an interference filter. All measurements were conducted using AC electric pulses with the characteristics summarized in Table 1.

#### 2.2.2. Procedure

Samples were aligned with the optical axis at an angle of $\pm \frac{\pi }{4}$ with respect to the crossed polarizers. Initially, each sample was brought to the isotropic phase (45 °C) and then cooled at a rate of 0.1 °C/min. The temperature $T^{**}$ was defined as the point where the sample began to exhibit non-zero transmitted intensity, indicating the onset of the nematic phase. This corresponded to a non-dark region in our camera recording as well. We defined $T^{*}$ as the temperature where all of the recorded images were non-dark, i.e., the whole sample was in the nematic phase.

During measurements in the nematic phase, once the temperature stabilized to within ±10 mK, the electric field was applied using the Python script. After each measurement, the sample was cooled to the next target temperature at the same rate. The smectic-A transition temperature, $T_{na}$, was identified by the sudden decrease in the flickering effect and confirmed by the absence of an electro-optical response due to the high elastic constants of the smectic phase.

#### 2.2.3. Dynamics

**Figure 2:**
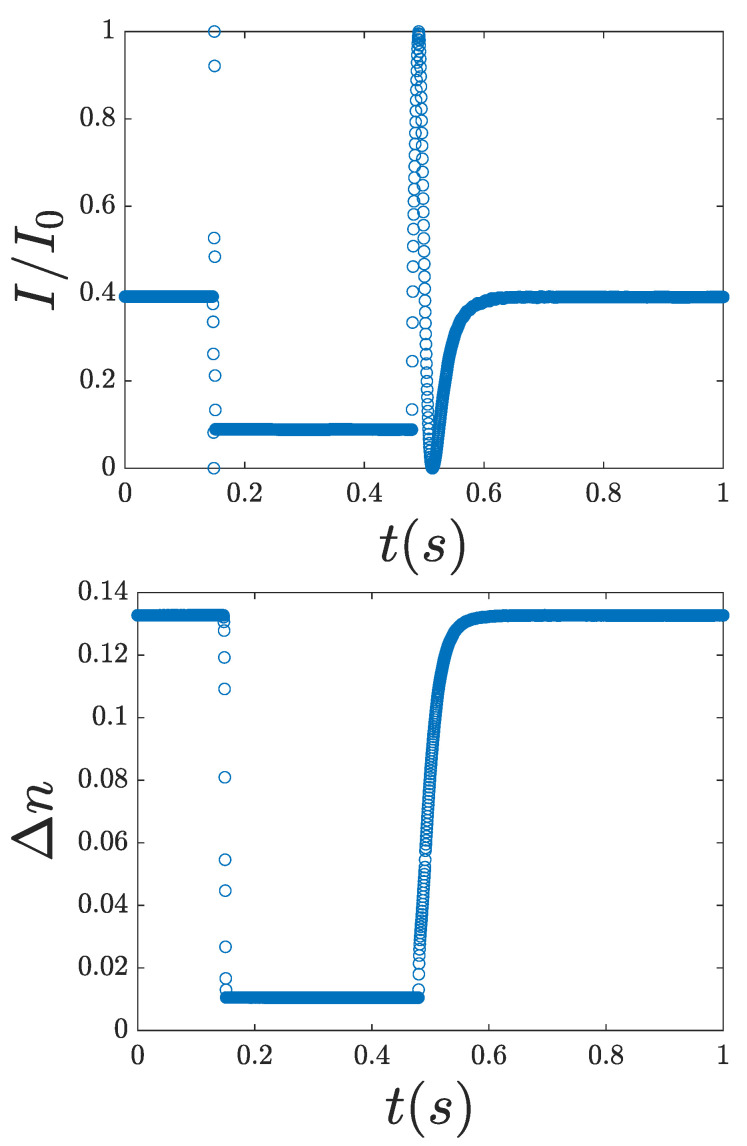
(**Top**) Normalized intensity. (**Bottom**) Birefringence. The electric field pulse is turned on at 0.1464 s and turned off at 0.4794 s.

As shown in Figure 2, the birefringence decreases upon application of voltage due to molecular reorientation and increases again when the voltage is removed. When the response time $\tau_{on}$ and relaxation time $\tau_{off}$ are shorter than the pulse and post-pulse acquisition duration, respectively, the birefringence reaches equilibrium. This allows the use of standard liquid crystal display characterization norms to define the switching times, namely, the 10–90% or 90–10% norms for $\tau_{on}$ and $\tau_{off}$, respectively. We estimated their values in three different ways: the *falltime* and *risetime* functions of Matlab, linear interpolation, and the same functions in OriginLab. In every analysis, the values differed by less than two times the step time, so we decided to use Matlab’s functions for code simplicity. The differences were due to the different methods of interpolation between the data points; assuming a different slope for fitting, the points of interest are positioned at different time values. Ultimately, for the estimation of the response and the relaxation times, we chose to use the *falltime* and *risetime* functions, respectively.

#### 2.2.4. Data Acquisition and Analysis

Each set of measurements from the oscilloscope contains 2500 data points. Homemade scripts were developed to streamline the data acquisition and analysis and to examine the response of the samples at different temperatures and with different amplitudes of applied voltage. A Python script was used to apply a sequence of AC pulses with varying amplitudes once the temperature stabilized within ±10 mK. Pulses with $V_{rms}$ ranging from 0.2 V to 5 V were applied. The optical response of each sample was recorded through the oscilloscope, printing each set of 2500 points of the transmitted light intensity in a file. A Matlab script was developed to semiautomate the analysis of the transmitted light, find the extrema, smooth, normalize, and convert the intensity to birefringence. The error bars for the birefringence data are equal to or smaller than the diameter of the data points in the plots.

## 3. Experimental Results

### 3.1. Microstructure, Phase Transitions, and Birefringence

**Figure 3:**
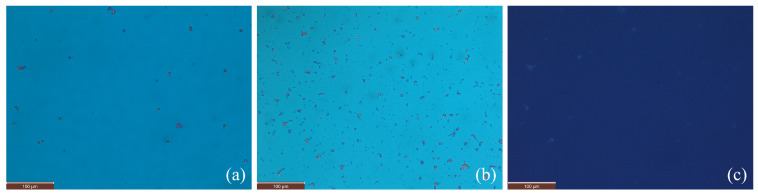
Sample microstructure observed under crossed polarizers using POM: (**a**) sample χ=0.2%; (**b**) sample χ=4.7%. (**c**) Fluorescence image of the χ=0.2% sample showing a homogeneous dispersion of carbon quantum dots.

Figure 3 presents polarized optical microscopy (POM) and fluorescence microscopy plates of representative samples in planar cells. The POM images, taken with crossed polarizers, correspond to samples with χ=0.2% (a) and χ=4.7% (b), while panel (c) shows the fluorescence image for χ=0.2%. The fluorescence image indicates a homogeneous dispersion of CQDs at χ=0.2%. The intensity histogram of the fluorescence image is presented in Figure S3. The POM image for χ=0.2% exhibits a uniform director field and no visible defects, indicating good alignment and homogeneous dispersion of CQDs. In contrast, the POM image for χ=4.7% reveals signs of CQD aggregation, suggesting a degradation of alignment quality at higher concentrations.

**Figure 4:**
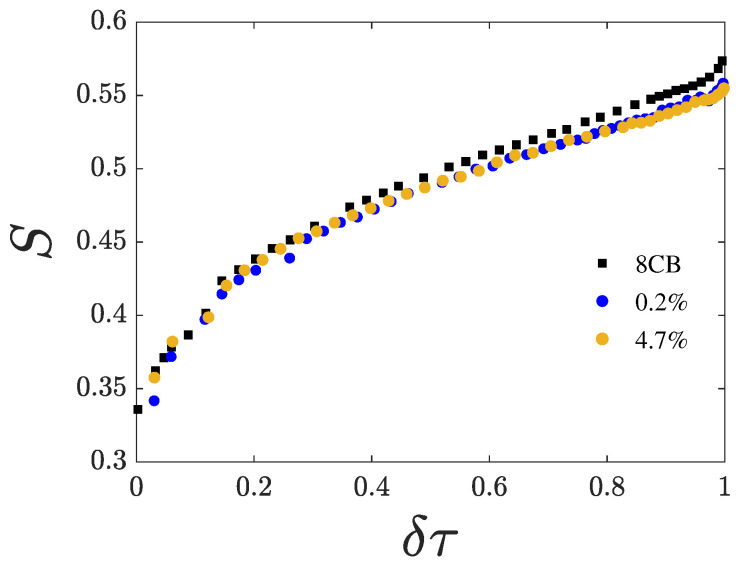
Nematic orientational order *S* as a function of the reduced temperature δτ. Pure 8CB, black symbols. Nanocomposites of CQDs in 8CB: 0.2% (blue symbols) and 4.7% (orange symbols).

We first measured the dependence of the phase transition temperatures $T_{in}$ and $T_{na}$ on the CQD mass ratio χ. The results are summarized in Table 2. Both $T_{in}$ and $T_{na}$ decrease with increasing χ, while the coexistence range of the isotropic–nematic transition broadens and the nematic range narrows. The birefringence of the samples was measured as a function of temperature, and Figure 4 shows *S* plotted against the reduced temperature defined as

(1) $$\delta \tau =\frac{T-T_{in}}{T_{na}-T_{in}}$$

for χ=0, 0.2, and 4.7%. For χ=0.2 and 4.7%, *S* reduces compared to the pure compound. Notably, *S* is nearly identical for all samples near $T_{in}$. However, as the temperature approaches $T_{na}$, *S* increases more slowly in the samples containing CQDs than in the pure compound.

By fitting the experimental data for Δn(T) with the Haller expression,

(2) $$\Delta n\left(T\right)=\Delta n_{0}\;{\left(1-\frac{T}{T_{0}}\right)}^{\beta }$$

where $\Delta n_{0}$ represents the birefringence for S=1, we determined the exponent β for each sample (see Table 2). The exponent β shows a slight variation with CQD concentration.

### 3.2. Threshold Voltage

By applying a variable voltage *V* to the samples and recording the optical response, we estimated the threshold voltage $V_{th}$ for the reorientation transition, which is related to the splay elastic constant $K_{1}$ and the dielectric anisotropy $\varepsilon_{a}$ through the well-known relation

(3) $$\begin{array}{c} V_{th}=\pi \;\sqrt{\frac{K_{1}}{\varepsilon_{0}\varepsilon_{a}}} \end{array}$$

where $\varepsilon_{0}$ is the vacuum permittivity. The criterion used to determine $V_{th}$ was the voltage at which the birefringence decreased to 90% of its initial value, measured in the absence of an electric field.

**Figure 5:**
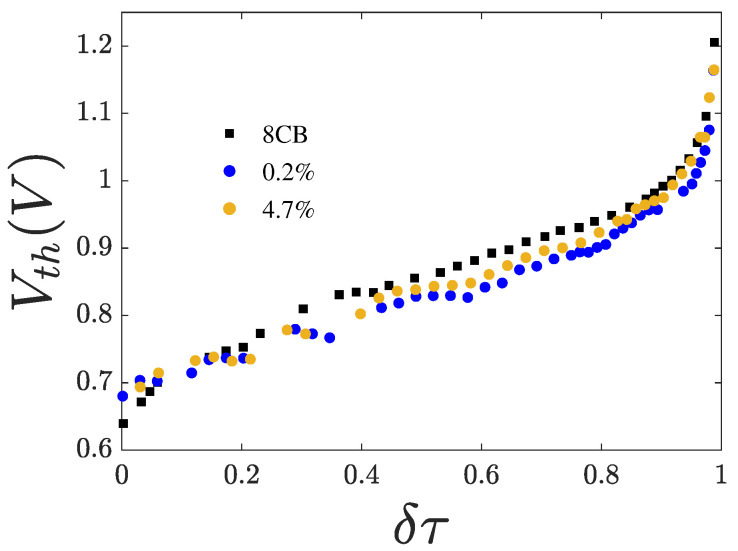
Iso-χ curves of the threshold voltage $V_{th}$ vs. reduced temperature δτ.

Figure 5 shows iso-χ curves of $V_{th}$ as a function of temperature. As shown, $V_{th}$ decreases with increasing χ. However, this reduction is not monotonic. The effect is most pronounced away from the phase transition temperatures, particularly in the middle of the nematic range. The observed decrease in the ratio $K_{1}/\varepsilon_{a}$ in the presence of CQDs suggests a softening of the elastic constant. Conversely, the increase in $V_{th}$ upon cooling reflects the stiffening of the elastic constant, which grows faster than the dielectric anisotropy. The divergence of $V_{th}$ near $T_{na}$ is discussed in Section 4.

### 3.3. Switching Times

**Figure 6:**
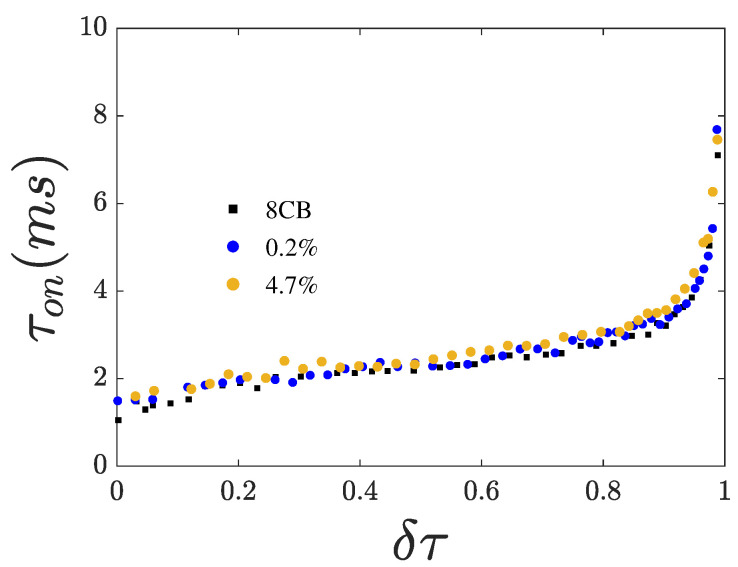
Response time $\tau_{on}$ vs. reduced temperature δτ for an impulse with *rms*-amplitude of 5 V.

**Figure 7:**
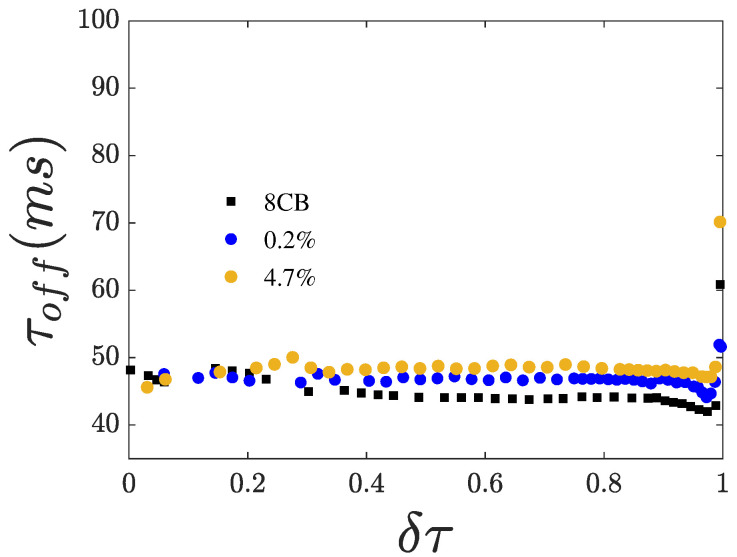
Relaxation time $\tau_{off}$ vs. reduced temperature δτ for an impulse with *rms*-amplitude of 5 V.

From the dynamics of the Fréedericksz transition, we extracted the switching times $\tau_{on}$ and $\tau_{off}$ shown in Figure 6 and Figure 7, respectively.

Both switching times increase with increasing χ, suggesting that the presence of CQDs leads to higher rotational viscosity. Both switching times diverge upon approaching $T_{na}$. Notably, $\tau_{off}$ shows a minimum close to $T_{na}$ just before the divergence. This minimum becomes less profound with increasing χ. The divergence of the switching times upon cooling toward $T_{na}$ reflects the corresponding divergence of the rotational viscosity $\gamma_{1}$.

## 4. Analysis

**Figure 8:**
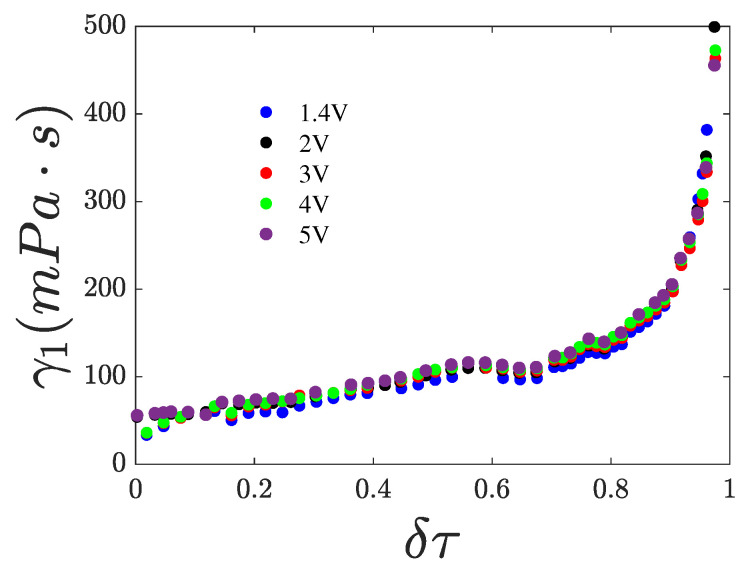
Pure 8CB, rotational viscosity extracted from $\tau_{off}$ vs. reduced temperature δτ for various voltage magnitude.

From the measured switching times and threshold voltage values, assuming that backflow effects are negligible, the rotational viscosity $\gamma_{1}$ can be calculated using the following relations:

(4) $$\begin{array}{cc} \tau_{on}= & \frac{\gamma_{1}d^{2}}{\varepsilon_{0}\varepsilon_{a}\left(V^{2}-V_{th}^{2}\right)} \end{array}$$

(5) $$\begin{array}{cc} \tau_{off}= & \frac{\gamma_{1}d^{2}}{\pi^{2}K_{1}} \end{array}$$

if the elastic constant and the dielectric anisotropy are known. It is important to note that for $V>>V_{th}$, backflow effects become non-negligible [38]. In this regime, the rotational viscosity in the switching time expressions is effectively replaced by an effective viscosity, and the splay elastic constant is substituted by an effective elastic constant that approaches the bend elastic constant at sufficiently high voltages. In our experiments, the maximum applied *rms* voltage was 5 V. In contrast, values up to 10 V are commonly reported in the literature, often without explicitly accounting for backflow effects or by assuming that their influence can be adequately described by an effective viscosity $\gamma_{1}^{*}=\gamma_{1}^{*}\left(V\right)$ dependent on the voltage magnitude. For simplicity, we henceforth omit the superscript * from the symbol $\gamma_{1}^{*}$ denoting the effective viscosity. To evaluate the role of backflow effects in our measurements, we plotted $\gamma_{1}$ as a function of the reduced temperature, calculated from the measured $\tau_{off}$, as shown in Figure 8. The different iso-field curves follow the same trend and nearly overlap. Considering that backflow effects are even less significant for $\tau_{on}$, we neglected any corrections due to backflow in our analysis by introducing an effective viscosity.

**Figure 9:**
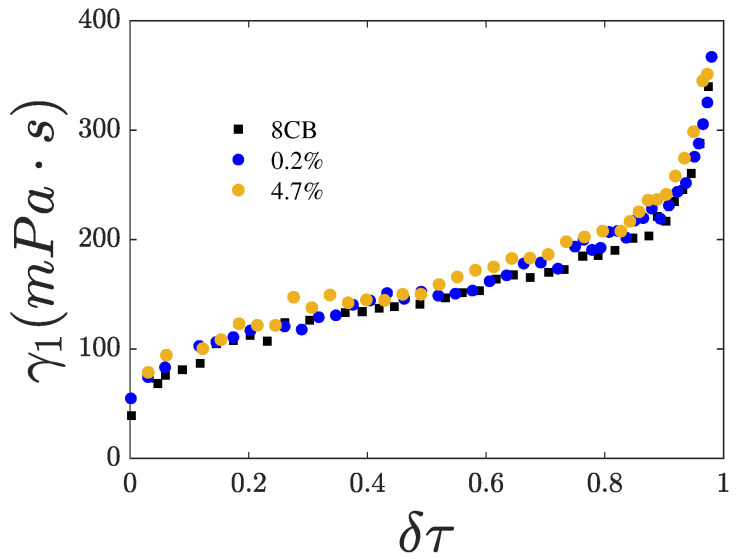
Iso-χ curves of rotational viscosity vs. reduced temperature, calculated from $\tau_{on}$ data.

Figure 9 shows the nematic orientational viscosity $\gamma_{1}$ as a function of the reduced temperature for different χ values of the doped samples. $\gamma_{1}$ is calculated from Equation (4) using the measured switch-on time. Elastic constants and dielectric anisotropy values were taken from [32,33]. $\gamma_{1}$ increases with cooling and diverges toward $T_{na}$.

### 4.1. Activation Energy

The activation energy $E_{a}$ informs us about the energy barrier for molecular rotation and depends on the temperature *T* and orientational order *S*. The simplest model does not consider any dependence of $\gamma_{1}$ on the order parameter. Within the framework of more elaborate models [17], the dependence of $E_{a}$ on *S*, as well as the dependence of $\gamma_{1}$ on *S*, is also taken into account. To introduce a compact expression for the activation energy, we define $E_{a}=\epsilon S^{y}$, where y=0, 1, 2, and ϵ is a constant representing the activation energy when S=1. Additionally, to describe the dependence of $\gamma_{1}$ on *S*, we introduce the notation $\gamma_{1}∼S^{x}$ with x=0, 1, 2. For x=0, $\gamma_{1}$ is apparently independent of *S*. While the case x=2 is thermodynamically justified [1], the case x=1 is often observed in nematic liquid crystals composed of mesogenic molecules with elongated conjugated chains [9].

**Figure 10:**
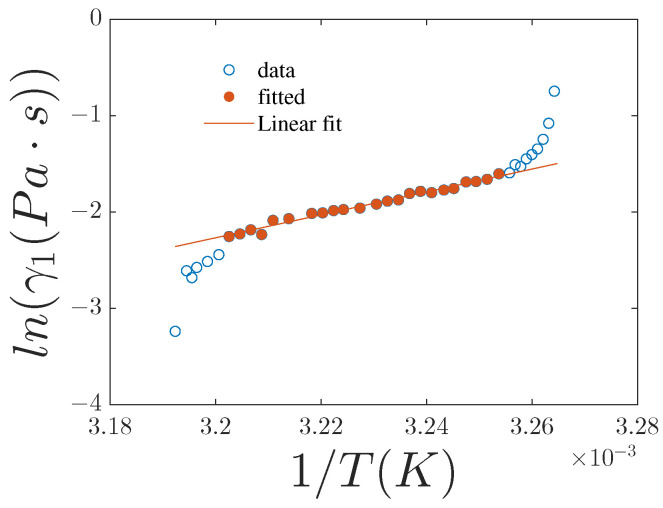
Plot of $\ln(\gamma_{1})$ vs. 1/T. Experimental data are shown as points. The red line represents the best linear fit calculated using the solid red data points.

An Arrhenius-type plot, far from both transition temperatures, of $\gamma_{1}$ as a function of 1/T, case y=0, and x=0, gives a straight line, as shown in Figure 10. The extracted activation energy is approximately 1 eV (see Table 3).

**Figure 11:**
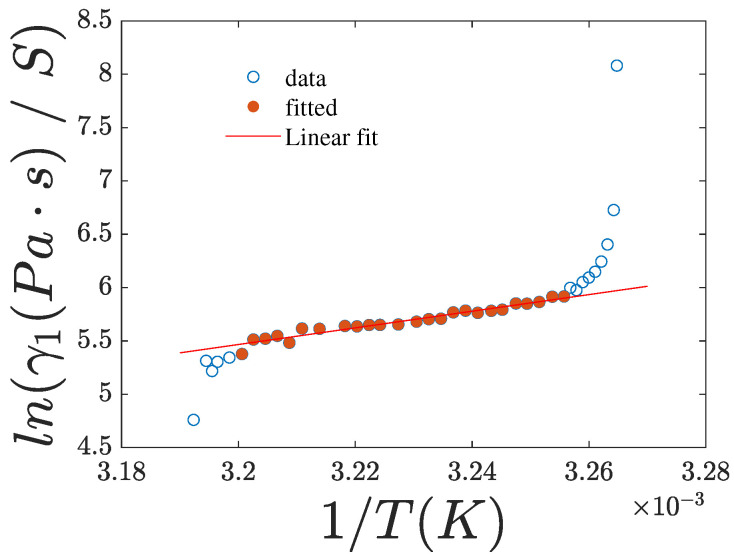
Plot of $\ln(\gamma_{1}/S)$ vs. 1/T. Experimental data are shown as points. The red line represents the best linear fit calculated using the solid red data points.

Figure 11 shows the plot of $\ln(\gamma_{1}/S)$ versus the inverse temperature 1/T (case x=1,y=0), where $E_{a}$ is assumed to be constant and independent of *S*, while $\gamma_{1}∼S$. The slope of the linear fit gives $E_{a}=0.67\;\mathrm{eV}$, which is in agreement with the measurement in [39], where a value of $E_{a}=0.74\;\mathrm{eV}$ was obtained for pure 8CB.

**Figure 12:**
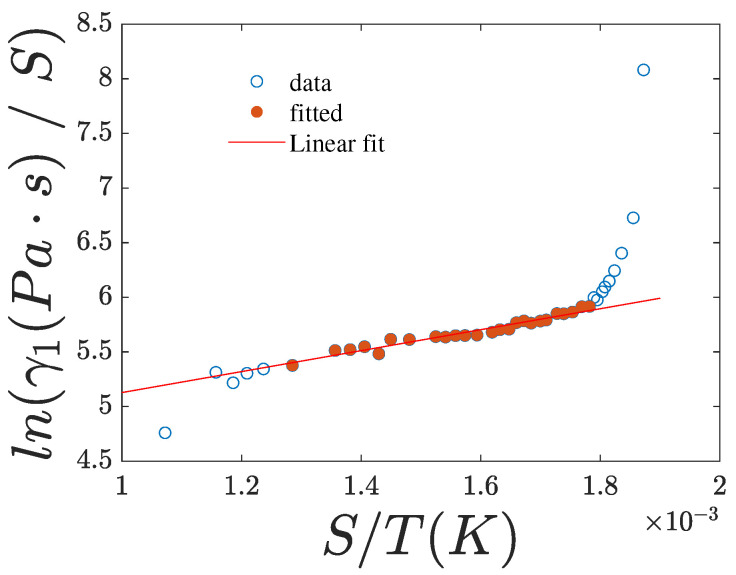
Plot of $\ln(\gamma_{1}/S)$ vs. S/T. Experimental data are shown as points. The red line represents the best linear fit calculated using the solid red data points.

A graph of $\ln(\gamma_{1}/S^{2})$ versus the ratio S/T (case x=2,y=1) also yields a straight line, from which the Maier and Saupe (MS) maximum interaction energy, $\epsilon =1.11\;k_{B}T_{in}$, is calculated. This value is rather low compared to the expected MS interaction potential. Nevertheless, when plotting $\ln(\gamma_{1}/S)$ against the ratio S/T, as shown in Figure 12, ϵ is estimated to be $3.08\;k_{B}T_{in}$, which is lower than the value of $4.29\;k_{B}T_{in}$ reported in [23] for 5CB. However, this lower value is expected due to the higher length-to-breadth ratio of 8CB with respect to 5CB [23].

Finally, in Table 3 are listed the obtained fitting values of ϵ for various models, for pure 8CB and doped samples. It is observed that, in all cases, ϵ increases with χ, while a linear dependence of $\gamma_{1}$ on *S* provides a better fit quality than a quadratic dependence.

### 4.2. Fitting of $\gamma_{1}\left(T,\chi \right)$

**Figure 13:**
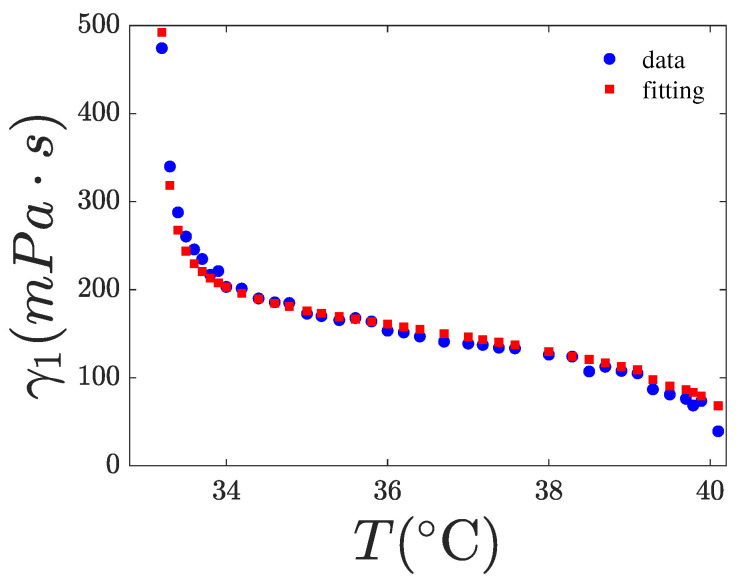
Rotational viscosity of 8CB vs. temperature. The red points are a fitting of the experimental data (blue) using Equation (6). The fitting results are tabulated in Table 4. Range of fit: 33.2–40.1 °C.

**Figure 14:**
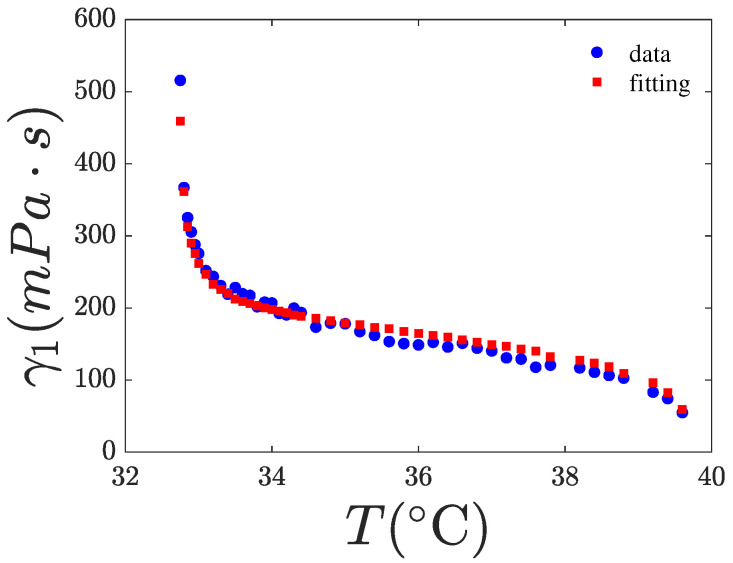
Rotational viscosity of the 8CB with χ=0.2% in CQDs vs. temperature. The red points are a fitting of the experimental data (blue) using Equation (6). The fitting results are tabulated in Table 4. Range of fit: 32.7–39.6 °C.

**Figure 15:**
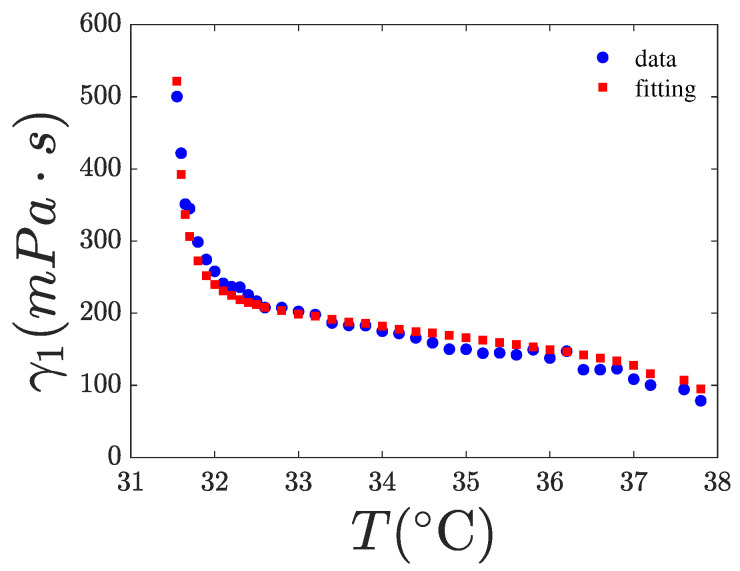
Rotational viscosity of the 8CB with χ=4.7% in CQDs vs. temperature. The red points are a fitting of the experimental data (blue) using Equation (6). The fitting results are tabulated in Table 4. Range of fit: 31.55–37.8 °C.

To fit our results on $\gamma_{1}$ in the entire nematic temperature range, we used the following equation:

(6) $$\gamma_{1}=g\;Exp\left(\frac{E_{a}}{k_{B}T}\right)\;S^{x}\;Exp\left(\frac{\theta \;S^{2}}{T-T_{f}}\right)$$

where $k_{B}$ is the Boltzmann constant, $T_{f}$ is the nematic fluctuation “freezing” temperature, and θ and *g* are fitting parameters considered as constants with respect to the temperature. The exponent *x* must be determined by fitting the experimental data. In the Diogo and Martins model [8], $E_{a}$ is a function of *S*, specifically given by $E_{a}=\epsilon S$ (y=1 case), representing the MS interaction potential. The last exponential in Equation (6) models the freezing of nematic director fluctuations and can be disregarded in the nematic phase if the temperature is sufficiently higher than $T_{f}$. For 8CB, the latter temperature is slightly lower than $T_{na}$. However, as for 8CB, $T_{na}$ and $T_{in}$ are only about 7 K apart, the influence of the last exponential term in Equation (6) remains significant even at temperatures close to $T_{in}$. The expression of $\gamma_{1}$ in Equation (6) contains five fitting parameters, making it difficult to accurately fit the $\gamma_{1}$ data without additional information to constrain some of them. Our fittings indicate that ϵ tends toward zero, suggesting that the first exponential term is effectively constant. This observation is consistent with the findings of [20] and with the theoretical prediction of the Diogo and Martins model [8]. This result may be interpreted as the dominance of the free volume factor over the MS factor in Equation (6). Therefore, we set ϵ=0 in our fittings, the results are shown in Figure 13, Figure 14 and Figure 15. Furthermore, we assumed that the parameters *g* and θ are independent of χ and, therefore, used the values obtained for pure 8CB to fit the data for the nanocomposites. Our best-fit parameters are listed in Table 4. As observed, the exponent of *S* in the power law is x=2 for the pure system and decreases with increasing χ, indicating that the presence of CQDs alters the power-law dependence of $\gamma_{1}$ on *S*. Notably, we fitted the rotational viscosity with the experimental data of both *T* and *S*. That means that we did not fit a curve, but we fitted our data with a 2D surface in 3D space, $\gamma_{1}\left(T,S\right)$. To validate our choice, we also fitted the experimental data of *S* as a function of *T* to create a smooth curve S(T). The curve’s function was used to fit the rotational viscosity to a line in a 2D space, $\gamma_{1}\left(T,S\left(T\right)\right)=\gamma_{1}\left(T\right)$. The estimated values of the free parameters $x,\;T_{f},\;\theta ,\;g$ from the latter fit coincide with the corresponding values obtained from the surface fit.

## 5. Discussion

At this point, we must comment on previous work measuring the rotational viscosity coefficient, $\gamma_{1}$, in pure liquid crystalline compounds and binary mixtures [39,40,41,42,43]. In [40], pure 8CB and mixtures of 8CB with biphenyl were investigated. It was found that $\gamma_{1}$ diverges as $T_{na}$ is approached from the nematic phase. Additionally, no anomalous behavior was observed for either the dielectric anisotropy or the splay elastic constant, both of which remain approximately constant at the nematic-to-smectic-A phase transition for all concentrations. Binary mixtures of mesogens have also been investigated [42,43]. In [42], a binary system composed of calamitic and hockey-stick-shaped mesogens was investigated, and the rotational viscosity seems to diverge while approaching $T_{na}$, while in [43], calamitic and bent-core mesogen mixtures were investigated, with no indication of divergence on approaching $T_{na}$. Nevertheless, in this latter work, the system probably did not approach close enough to $T_{na}$.

According to our measurements, a divergence of $\gamma_{1}$ is observed for all χ values used in our investigations. This divergence of the switching times and, hence, of $\gamma_{1}$ is due to the onset of the smectic short-range fluctuations in the nematic phase close to $T_{na}$, as discussed in [24]. Nevertheless, as shown in Figure 7, the relaxation time $\tau_{off}$ in cooling initially shows a decrease followed, on further cooling, by a divergence. Since neither the dielectric anisotropy ($\varepsilon_{a}$) nor the splay elastic constant $K_{1}$ exhibit critical behavior at $T_{na}$, this acceleration of $\tau_{off}$ before slowing down when approaching $T_{na}$ from above should originate from a faster increase in an elastic constant than the increase in $\gamma_{1}$. This behavior suggests that the contribution of $K_{3}$, which is well known to diverge near $T_{na}$, is significant. That is, the elastic deformation is no longer purely splay, and the bend contribution becomes important. Finally, the local minimum of $\gamma_{1}$ becomes shallower in the presence of CQDs, indicating that the divergence of $K_{3}$ is softened in the presence of CQDs.

Concerning the puzzling question of the activation energy, we have shown that in order to fit the entire nematic temperature range, the best fits are obtained when $E_{a}$ is treated as a constant, and $\gamma_{1}∼S^{x}$ is used with x=2 for pure 8CB. On the other hand, if one keeps only data far from both transition temperatures, then fitting with $E_{a}=\epsilon S$ (x=1) gives a value for the MS interaction potential of $3.08\;k_{B}T_{\mathrm{in}}$, which is lower than the theoretically predicted value of $4.55\;k_{B}T_{\mathrm{in}}$; nevertheless, it is in good agreement with the measured values reported in [23]. Of course, other possibilities for the fitting function exist, as discussed in detail in [17]. Some of these are presented in Table 4. However, the main difficulty in using more elaborate models lies in the increasing number of fitting parameters, which can lead to the emergence of multiple, practically equivalent minima. In any case, our main aim in this work was not the validation of a particular model but merely the investigation of the influence of doping the LC compound with CQDs on the rotational viscosity. Finally, our experimental results show that the particular CQDs used for doping 8CB have a limited impact on $\gamma_{1}$, $E_{a}$, and the switching times, while a decrease of up to 8% in $V_{th}$ was measured.

Certainly, other nanocomposites should be studied to gain a clearer understanding of the impact of nanoparticles (NPs) on the rotational viscosity of the nematic phase, particularly in the vicinity of a nematic-to-smectic-A transition. Based on our experience and the literature [44,45,46,47,48,49], we know that other types of NPs dispersed in a liquid crystalline matrix (LCM) can have a significantly stronger impact on the properties of the LCM. Investigating the influence of other types of NPs on the rotational viscosity coefficient $\gamma_{1}$ and $E_{a}$ is, therefore, both interesting and part of our future research plans.

## 6. Conclusions

We measured the birefringence, phase transition temperatures, Fréedericksz-threshold voltage, and switching times of nematic cells in splay geometry of pure and doped 8CB with CQDs. The analysis of these measurements gave us access to the order parameter, the activation energy, and the rotational viscosity in the nematic phase. Through this work, we have shown that (i) switching times become longer with concentration; (ii) the order parameter slightly decreases with χ; (iii) $V_{th}$ behaves non-monotonically with χ—it first decreases, but for higher χ values, it increases; however, it always remains lower than $V_{th}$ for the pure system; (iv) $\gamma_{1}$ and $V_{th}$ diverge when cooling towards the smectic phase, regardless of the concentration of CQDs; (v) the fitting of the dependence of $\gamma_{1}$ on $S^{x}$ gives an exponent x=2 for the pure compound, and *x* decreases with χ; (vi) the activation energy calculated by fitting our data in the entire nematic temperature range is constant; and (vii) the Maier and Saupe potential can be calculated by fitting only the central part of the data far from both transition temperatures.

## Figures and Tables

**Table 1:** Characteristics of the applied pulses.

Characteristic	Value
Shape	Square
Function	Sinusoidal
Frequency	1 KHz
Voltage $V_{rms}$	0–5 V
Duration of pulse	0.333 s
Step of acquisition	0.4 ms
Duration of acquisition	1 s

**Table 2:** Phase transition temperatures and exponent β of the samples as a function of composition χ.

	8CB	0.2% *w*/*w*	4.7% *w*/*w*
β	0.155 ± 0.005	0.1604 ± 0.0006	0.1628 ± 0.0007
$T^{**}\;{(}^{\circ }C)$	40.17	39.85	38.74
$T_{in}\;{(}^{\circ }C)$	40.12	39.61	38.0
$T_{na}\;{(}^{\circ }C)$	33.12	32.66	31.47
Nematic Range ${(}^{\circ }C)$	7.00	6.95	6.53

**Table 3:** Fitting results for different models as a function of χ.

Fit Function	8CB		0.2% *w*/*w*		4.7% *w*/*w*	
	ϵ (**eV**)	$R^{2}$	ϵ (**eV**)	$R^{2}$	ϵ (**eV**)	$R^{2}$
ln($\gamma_{1}/S$) vs. 1/T	0.67 ± 0.07	0.944	0.70 ± 0.09	0.917	0.76 ± 0.10	0.909
ln($\gamma_{1}/S$) vs. S/T	0.083 ± 0.008	0.947	0.092 ± 0.015	0.879	0.107 ± 0.017	0.877
ln($\gamma_{1}/S^{2}$) vs. 1/T	0.25 ± 0.07	0.721	0.31 ± 0.10	0.637	0.41 ± 0.11	0.707
ln($\gamma_{1}/S^{2}$) vs. S/T	0.030 ± 0.009	0.679	0.040 ± 0.015	0.561	0.056 ± 0.018	0.652
ln($\gamma_{1}$) vs. S/T	0.135 ± 0.008	0.981	0.144 ± 0.014	0.95	0.157 ± 0.017	0.943
ln($\gamma_{1}$) vs. $S^{2}/T$	0.145 ± 0.008	0.982	0.155 ± 0.014	0.956	0.166 ± 0.017	0.949
ln($\gamma_{1}$) vs. 1/T	1.03 ± 0.06	0.982	1.12 ± 0.07	0.975	1.11 ± 0.09	0.965

**Table 4:** Fitting results using Equation (6) as a function of χ for ϵ=0.

Parameters	8CB	0.2% *w*/*w*	4.7% *w*/*w*
Range (°C)	33.2–40.1	32.7–39.6	31.6–37.8
*T_f_* (°C)	33.07	32.61	31.43
*g* (mPa·s)	601 ± 16	601	601
θ (°C)	0.375 ± 0.021	0.375	0.375
χ	2 ± 0.02	1.86 ± 0.03	1.80 ± 0.04
$R^{2}$	0.983	0.985	0.97

## Data Availability

The research data of this paper are available upon request.

## Supplementary Materials

The following supporting information can be downloaded at: https://www.mdpi.com/article/10.3390/nano15161278/s1, Figure S1: ^1^H NMR spectra of alkyl-functionalized CDs; Figure S2: Fluorescence spectra of alkyl-functionalized CDs. Figure S3: The fluorescence intensity distribution over the surface of the CQDs.
