# Supplementary material for: Influence of Carbon Quantum Dots on the Orientational Order and Rotational Viscosity of 8CB
Source: Nanomaterials (Basel). 2025 Aug 19;15(16):1278. doi: 10.3390/nano15161278 (PMC12389076; doi:10.3390/nano15161278)
Supplement: Supplementary file 1 [file nanomaterials-15-01278-s001.zip › nanomaterials-3805956-supplementary.pdf]

Influence of Carbon Quantum Dots on the Orientational Order and Rotational Viscosity of 8CB

Alfredos Schinas, Stefanos Basim Atata, Dimitris Tsiourvas and Ioannis Lelidis

Supplementary Materials

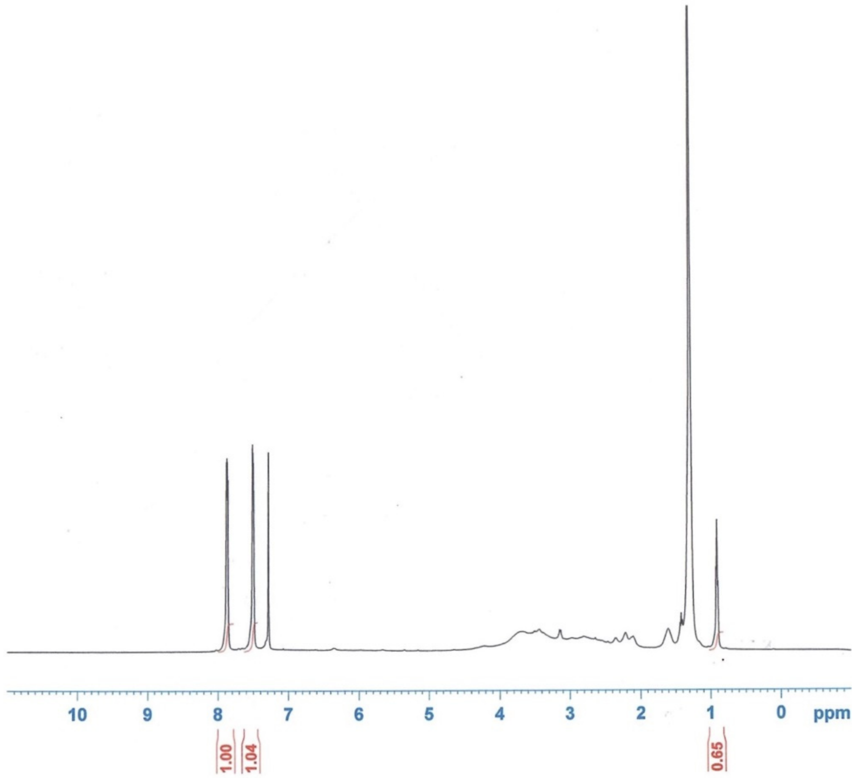

Figure S1. ^{1}H NMR spectra of alkyl-functionalized CDs in MeOD employing naphthalene as an internal standard.

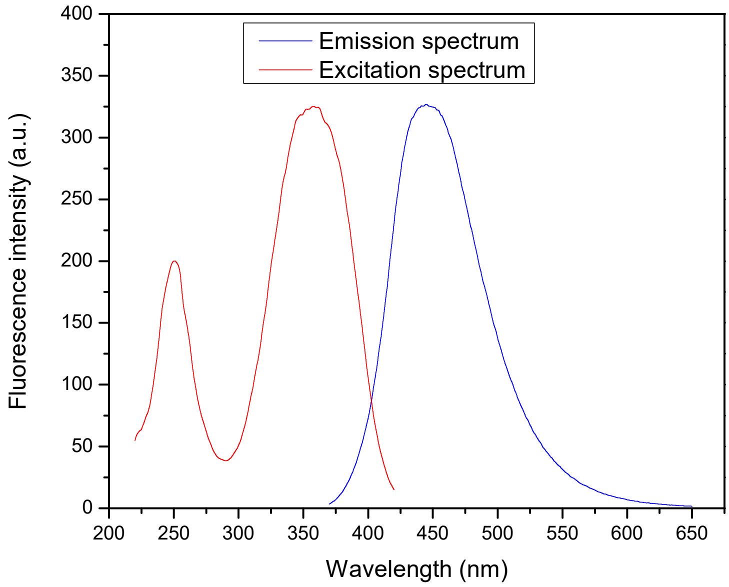

Figure S2. Excitation (red line, λ_{em} = 446 nm) and emission spectra (blue line, λ_{ex} = 356 nm) of alkyl-functionalized CDs in ethanol.

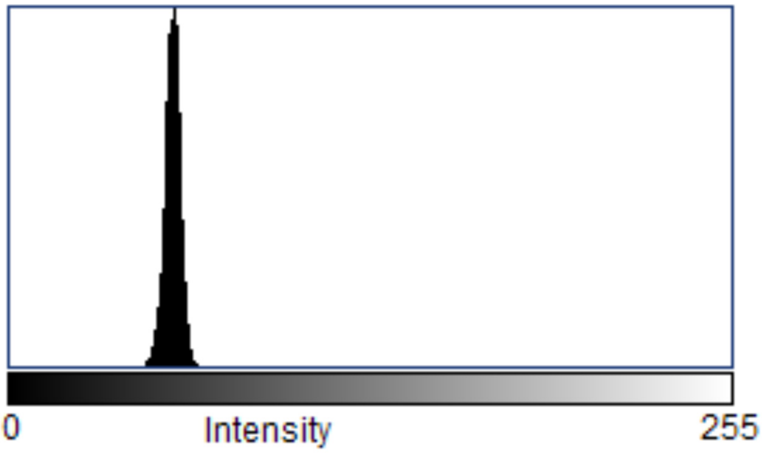

Figure S3 The fluorescence intensity distribution over the entire surface shown in Figure 3c corresponds to the sample with χ = 0.2% w/w, excited at 365 nm. The distribution has a mean intensity of 57.3 and a standard deviation of 2.9.
